# FTO attenuates the cytotoxicity of cisplatin in KGN granulosa cell-like tumour cells by regulating the Hippo/YAP1 signalling pathway

**DOI:** 10.1186/s13048-024-01385-5

**Published:** 2024-03-15

**Authors:** Rongli Wang, Feiyan Cheng, Xinyuan Yang

**Affiliations:** 1 Department of Obstetrics and Gynecology, Peking University People’s Hospital, No. 11, Xi-Zhi-Men South Street, Xi Cheng District, Beijing, 100044 China; 2 Department of Obstetrics and Gynecology, First Affiliated Hospital, Xi’an Jiaotong University, Xi’an, 710061 China

**Keywords:** Fat mass- and obesity-associated, Hippo/YAP1, Granulosa, Apoptosis, Premature ovarian failure

## Abstract

Premature ovarian failure (POF) is a devastating condition for women under 40 years old. Chemotherapy, especially the use of cisplatin, has been demonstrated to promote the apoptosis of granulosa cells in primary and secondary follicles, leading to POF. Our previous studies demonstrated that fat mass- and obesity-associated (FTO) plays an essential role in protecting granulosa cells from cisplatin-induced cytotoxicity. Various studies have suggested that the Hippo/YAP signalling pathway plays a significant role in regulating cell apoptosis and proliferation. Additionally, YAP1 is the main downstream target of the Hippo signalling pathway and is negatively regulated by the Hippo signalling pathway. However, whether the Hippo/YAP signalling pathway is involved in the protective effect of FTO on granulosa cells has not been determined. In this study, we found that after cisplatin treatment, the apoptosis of granulosa cells increased in a concentration-dependent manner, accompanied by the downregulation of FTO and YAP1. Furthermore, overexpression of FTO decreased cisplatin-induced granulosa cell apoptosis, inhibited the Hippo/YAP kinase cascade-induced phosphorylation of YAP1, and promoted the entry of YAP1 into the nucleus. The downstream targets of YAP1 (CTGF, CYR61, and ANKRD1) were also increased. Si-RNA-mediated downregulation of FTO promoted cisplatin-induced granulosa cell apoptosis, activated the Hippo/YAP kinase cascade, and inhibited the YAP1 entry into the nucleus. These effects were completely reversed by the small molecule inhibitor of YAP1-verteporfin (VP). Taken together, these data suggested that FTO-YAP1 plays a positive role in regulating the proliferation of injured granulosa cells induced by cisplatin.

**Supplementary Information:**

The online version contains supplementary material available at 10.1186/s13048-024-01385-5.

## Introduction

Follicles in mammalian ovaries are composed mainly of the oocytes, theca cells, and granulosa cells [1]. In an individual follicle, granulosa cells lie outside the zona pellucida and provide nutrients and growth factors for the oocytes and theca cells [2]. Premature ovarian failure (POF) is a reproductive endocrine disease, and the key features of POF are hormonal deficits and infertility, which can be devastating for women of childbearing age [3]. It has many causes, and its pathogenesis is complex. The most important factor is the acceleration of follicular atresia. In atretic follicles, granulosa cells undergo earlier apoptosis than oocytes and theca cells, suggesting that granulosa cells play an important role in the initiation of follicular atresia [1]. Furthermore, by regulating the expression of apoptosis-related genes, such as Fas / FasL [4], TNF - α [5, 6], Trail [7], and Bcl-2 family [8], in granulosa cells, we can accurately regulate the growth of follicles and the occurrence of follicle atresias. Taken together, these findings suggest that the proliferation and apoptosis of granulosa cells play important roles in the development of follicles and the maintenance of ovarian function.

Cisplatin, a widely used chemotherapeutic agent, is a first-line drug for the treatment of various solid cancers, including the head and neck, bladder, lung, lymphoma, melanoma, and several others [9]. However, the side effects caused by cisplatin treatment should not be ignored, especially reproductive toxicity for women of childbearing age [10, 11]. For example, gonadotoxicity induced by cisplatin changes the menstrual cycle and promotes the apoptosis of granulosa cells in primary and secondary follicles, resulting in follicle atresia, ovarian reserve depletion, and then POF [12, 13]. However, to date, there is no clear method for reducing the reproductive toxicity of cisplatin.

Fat mass- and obesity-associated (FTO) protein is the first discovered RNA demethylase and has been confirmed to be associated with obesity [14], metabolic syndrome [15], and diabetes risk [16]. Recent studies have identified other biological functions of FTO, such as promoting cell proliferation, inhibiting cell apoptosis, and increasing the cancer cell tolerance to chemotherapy [17–19]. In the ovaries of patients and animals with POF, the expression of FTO decreased significantly [20, 21]. Moreover, our previous study demonstrated that the overexpression of FTO in cisplatin-induced injured granulosa cells could reverse the proliferation of cells to that of negative control (NC) groups cells [21]. However, little is known about the mechanism by which FTO plays a protective role in cisplatin-induced injury.

The Hippo signalling pathway is highly conserved. In mammals, it consists of a kinase cascade. Activation of Hippo kinase cascades, such as Ste20-like kinases 1/2 (MST1/2) and large tumour suppressor 1/2 (LATS1/2) cascades [22, 23], induces phosphorylation of Yes-associated protein 1 (YAP1). Subsequently, YAP1 is restricted to the cytoplasm and degraded [24–26]. In contrast, inhibiting the Hippo signalling pathway, such as through blocking the MST1/2 and LATS1/2, promoted YAP1 translocation to the nucleus [27]. Only the overexpression and nuclear localization of the YAP1 protein was found to be involved in cell proliferation and survival. Various studies have demonstrated that YAP1 is an oncogene that plays a vital role in tumorigenesis via promoting cell proliferation [28, 29]. Recently, a growing number of studies have shown that YAP1 can promote the proliferation of ovarian granulosa cells and is involved in the development of follicles [23, 26, 30, 31]. Downregulation of YAP1 disrupted follicle development both in vitro and in vivo [26]. Furthermore, inhibiting the expression of YAP1 in granulosa cells promoted apoptosis, decreased the number of corpora lutea, and reduced the size of the ovaries, ultimately leading to the subfertility in mice [26]. A recent study indicated that the FTO/YAP1 axis plays an important role in oral squamous cell carcinoma (OSCC) cell proliferation [32]. However, the role of the FTO/YAP1 axis in the cisplatin-induced injury in granulosa cells has not been determined.

In the present study, we found that cisplatin induces granulosa cell cytotoxicity by downregulating the expression of FTO, activating the Hippo signalling pathway, and inhibiting the translocation of YAP1 into the nucleus. Overexpression of FTO significantly disrupted the Hippo signalling pathway and activated the YAP1 expression, which promoted cell proliferation and decreased cell apoptosis.

## Materials and methods

### Chemicals and antibodies used in this study

The culture medium of DMEM/F12 was purchased from Hyclone (Logan, UT). Fetal bovine serum (FBS) was obtained from Sijiqing (Zhejiang, China). The 0.25% trypsin-EDTA was purchased from Thermo Fisher Scientific (Waltham, MA, USA). The cisplatin was purchased from Sigma–Aldrich, St. Louis, MO and dissolved in 0.9% saline to a concentration of 1 mg/ml, stored at 4 °C in the dark. The FTO plasmid was designed and synthesized by Miaoling (Wuhan, China). siRNA aimed at FTO was synthesized by RiboBio (Guangzhou, China). Lipofectamine 2000 (Thermo Fisher Scientific, Waltham) was used for the following transfection according to the manufacturer’s instructions. The inhibitor of YAP1-verteporfin (VP) was purchased from MedChemExpress (Shanghai, China).

The primary antibodies include: FTO (1:1000, ab126205, Abcam, USA), MST1 (1:1000, 22245-1-AP, Proteintech, China), phosphor - MST1 (Thr183)/MST2 (Thr180) (E7U1D) (1:1000, 49,332, CST, USA), LATS1 (1:1000,17049-1-AP, Proteintech, China), phosphor - LATS1 (Ser909) (1:1000, 9157, CST, USA), YAP1 (D8H1X) (1:1000, 14,074, CST, USA), phospho -YAP (Ser127) (D9W2I) (1:1000, 13,008, CST, USA), BAX (1:1000, 50599-2-Ig, Proteintech, China), Bcl-2 (1:1000, 12789-1-AP, Proteintech, China), PCNA (1:1000, 10,205–2 – AP, Proteintech, China), β-actin (1:1000, 66099-1-Ig, Proteintech, China), cleaved-caspase 3 (1:1000, 9661S, CST, USA).

### The KGN cells culture and treatment

The KGN cell line (a human ovarian granulosa cell line) was purchased from *Procell Life Science&Technology Co., Ltd* (Wuhan, China). The cells were cultured in DMEM/F12 with 10% FBS at 37℃ in 5%CO_2_, and which were sub-cultured every other day. 2 × 10^5^ KGN cells were seeded per well in a six-well plate one day in advance. The following day, the culture medium was replaced, and 2 ml of complete medium was added to each well. Subsequently, 0, 0.6, 3, and 6 µl of cisplatin (1 mg/ml) were added to individual wells to achieve final concentrations of 0, 1, 5, and 10 µM respectively.

### FTO protein overexpression and knockdown

Overexpression of FTO was achieved by transfection the pCAG-FTO (human)-3×FLAG, and an empty vector (P19606/pCAG-MCS-3×FLAG) was used as a negative control (NC). FTO protein knockdown was performed with human FTO-specific small interfering RNAs (siRNAs) (Ribobio, Guangzhou, China). Among the 3 siRNAs, at least 2 of them with different sequences were verified to significantly knock down the expression level of FTO. Lipofectamine 2000 regent was used for the cells transient transfection. For the plasmid transfection, 3.0 µg vector DNA and 3 µL Lipofectamine 2000 were added to each well of the 6-well plate, when the cell confluence reached to 60–80%. And for the siRNA transfection, 50 nM siRNAs were transfected with 3 µL Lipofectamine 2000 per well for the 6-well plate. According to the manufacturer’s instruction, after transfected for 6 h, the serum free medium was replaced with complete culture medium. Finally, the transfection efficiency was confirmed by qRT-PCR and western blotting.

### Flow cytometry assay for cell apoptosis

KGN cells transfected with plasmid or siRNAs were harvested and stained with Annexin V-PE / 7-AAD according to the manufacturer’s instruction (BD Biosciences, CA, USA). In brief, after transfection, the KGN cells were cultured in the 6-well plate for another 48 h, and then harvested and centrifuged at 1000 rpm / min for 5 min. Washing the cells with ice – cold PBS for twice and resuspending them in 100 µL 1 × binding buffer (diluted with distilled water). Next, 5 µL PE-Annexin V and 5 µL 7-aminoactinomycin D (7-AAD) were added and incubated for another 15 min (at room temperature, in the dark). Finally, another 400 µL 1 × binding buffer added and flow cytometer (Benton Dickinson, Mountain view, CA) was used to measure and analyze the apoptosis rate.

### Western blotting analysis

Protein lysates from KGN cells were prepared by radioimmunoprecipitation assay buffer (RIPA) and the concentration of the lysates were determined by bicinchoninic acid (BCA) protein assay kit (Beyotime, Shanghai, China). A total of 30 µg protein per sample were added and separated by electrophoresis on 10% sodium dodecyl sulphate-polyacrylamide gel. Then, the proteins were transferred to the polyvinylidene difluoride membranes. Blocked the membranes with 5% nonfat milk, and incubated with primary antibodies at 4℃ overnight. The next day, incubated the membranes with the specific HRP-conjugated secondary antibody for 1 h at room temperature. All the images were visualized using an ECL kit (Millipore, MA). We measured the grayscale values of the target bands and their corresponding β-actin bands separately using Image J. Then, we calculated the ratio between the grayscale intensities of each target band and its internal reference. Finally, inter-group comparisons were conducted.

### RNA extraction and qRT-PCR assay

Total RNA was isolated from KGN cell line using Trizol reagent (Invitrogen, CO., Carlsbad, CA, USA), the concentration and purity of the RNA were detected, and complementary DNA (cDNA) was synthesisd as our previously described [33]. qRT-PCR was performed using SYBR Premix ExTaq™ (Takara) on the StepOne Real – Time PCR System (Applied Biosystems, USA) and the relative expression levels of the target genes were analyzed by the 2^−ΔΔCt^ method. The primers used in this study are as follows:

- GAPDH forward primer: 5’ - AAAATCAAGTGGGGCGATGCT − 3’
- GAPDH reversed primer: 5’ - TGGTTCACACCCATGACGAAC − 3’
- FTO forward primer: 5’ - CTT CAC CAA GGA GAC TGC TATTTC − 3’
- FTO reversed primer: 5’ - CAA GGT TCC TGT TGA GCACTCTG − 3’
- ANKRD1 forward primer: 5’- AGTAGAGGAACTGGTCACTGG − 3’
- ANKRD1 reversed primer: 5’- TGTTTCTCGCTTTTCCACTGTT − 3’
- CYR61 forward primer: 5’ - GGTCAAAGTTACCGGGCAGT − 3’
- CYR61 reversed primer: 5’ - GGAGGCATCGAATCCCAGC − 3’
- CTGF forward primer: 5’ - GGAAATGCTGCGAGGAGTGG − 3’
- CTGF reversed primer: 5’- GAACAGGCGCTCCACTCTGTG − 3’.

### Cell proliferation assay

The capacity of the KGN cells to proliferate was determined by a CCK – 8 assay kit (Dojindo, Kumamoto Prefecture, Japan). 1 × 10^4^ cells cultured in 100 µL complete medium were seeded in a 96-well plate (per well). After incubated for 24 h in the 37 ℃, at 5% CO_2_, 10 µL of CCK-8 reagent was added to each well and incubated for another 2 h. Then, the cells absorbance was evaluated at 450 nm using a microplate reader (Model 550; Bio-Rad, Shanghai, China).

### Immunofluorescence assay

Immunofluorescence was used to detect the expression and location of YAP1 in the KGN cells. KGN cells were seeded on coverslipes in a 6-well plate. After different treatment, the cells were washed twice with ice - cold PBS, then fixed with 4% paraformaldehyde for 0.5 h and permeabilized with 0.5% Triton X-100 (Beyotime) for 0.5 h at room temperature. Blocked the cells in 5% bovine serum albumin (BSA) for 1 h and incubated the cells with rabbit monoclonal antibody against YAP1 (1:200, Proteintech) at 4 ℃ overnight. The next day, washed the cells with PBS-T (0.1% Triton X-100/PBS) for three times, incubated the cells with anti-rabbit Alexa Fluo488 (Invitrogen) for 1 h in the dark, and incubated cells with 4’-diamidino-2-phenylindole (DAPI) for 0.5 h in the dark at room temperature. Finally, washed the cells with ice-cold PBS-T for 3 times and obtained the images using a fluorescence microscope (Olympus Inc., USA).

### Statistical analysis

All experiments were repeated at least 3 times. Statistical analysis was conducted using GraphPad Prism 5 (GraphPad Software, San Diego, California, USA). Data were presented in mean ± standard error of the mead (SEM). Student’s t-test and One-way ANOVA with Turkey’s post hoc test were applied to evaluate the Group difference.

## Results

### Cisplatin promoted KGN cell apoptosis and decreased the expression of FTO and YAP1

**Fig. 1:**
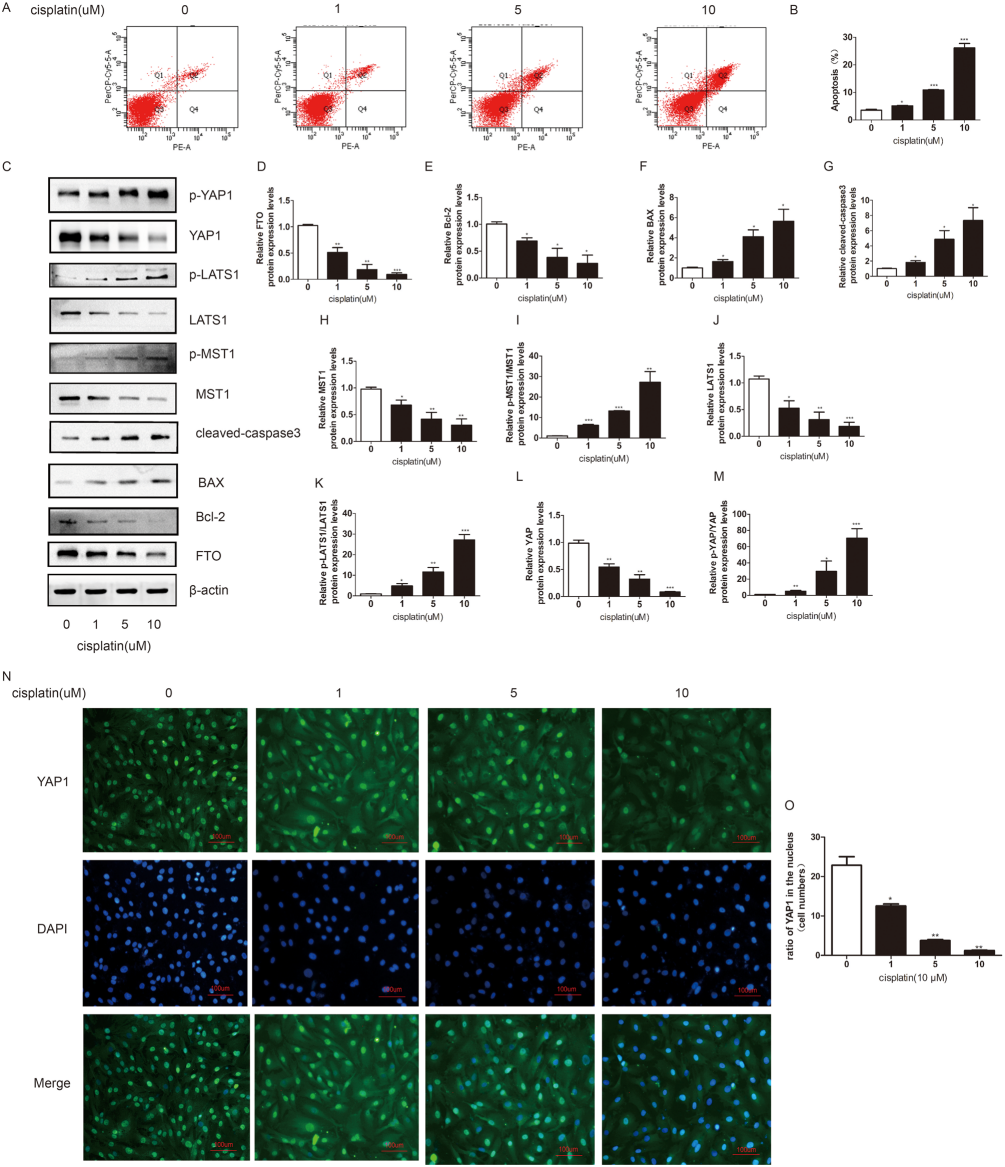
Cisplatin promoted the KGN cells apoptosis, decreased the expression of FTO, and activated the Hippo/YAP signaling pathway, in a dose dependent manner. (**A-B**) Flow cytometry assays were performed to determine the effect of cisplatin on KGN cells apoptosis. And a quantitative analysis of the rate of apoptosis was performed. (**C-M**) Western blot assay was applied to detect the effect of cisplatin on the protein expression level of apoptosis related protein, including Bcl-2, BAX, and cleaved-caspase-3, FTO and the key molecular of Hippo/YAP signaling pathway. β-actin was used as a loading control. (**N-O**) Immunofluorescence assay was performed to detect the effect of cisplatin on the expression and location of YAP1. (scale bar: 100 μm). Student’s t-test. The results were showed mean ± SEM from three independent experiments. (α = 0.05, *P < 0.05, **P < 0.01, ***P < 0.001)

To investigate the mechanism by which cisplatin affects KGN cell apoptosis,flow cytometry analysis was used, and the results showed that cisplatin promoted KGN cell apoptosis in a dose-dependent manner (0–10 µM) (Fig. 1A-B). Furthermore, a series of key proteins related to KGN cell survival were measured. Western blotting was performed for FTO, BAX, Bcl-2, cleaved-caspase3, MST1, LATS1, and YAP1 and for the ratio of p-MST1/MST1, p-LATS1/LATS1, and p-YAP1/YAP1. As shown in the Fig. 1C-M, with increasing concentrations of cisplatin, the protein expression level of FTO decreased significantly, and this change was accompanied by increases in the expression levels of BAX and cleaved-caspase3 and a decrease in the expression levels of Bcl-2. In addition, the expression of the YAP1 protein decreased significantly after the cisplatin treatment, whereas the relative ratio of p-YAP1/YAP1 was significantly increased. The kinase proteins of the Hippo signalling pathway, such as MST1 and LATS1, gradually decreased. The relative ratios of p-MST1/MST1 and p-LATS1/LATS1 were significantly increased. Consistent with the western blot results, immunofluorescence demonstrated that in cisplatin-induced injured KGN cells, the expression level of YAP1, which is mainly localized in the nucleus of proliferative granulosa cells, was downregulated significantly (Fig. 1N - O). These results indicated that FTO was downregulated or inhibited may in a Hippo/YAP signalling pathway-dependent manner after cisplatin treatment.

### FTO decreased KGN cell apoptosis by promoting the YAP1 expression

**Fig. 2:**
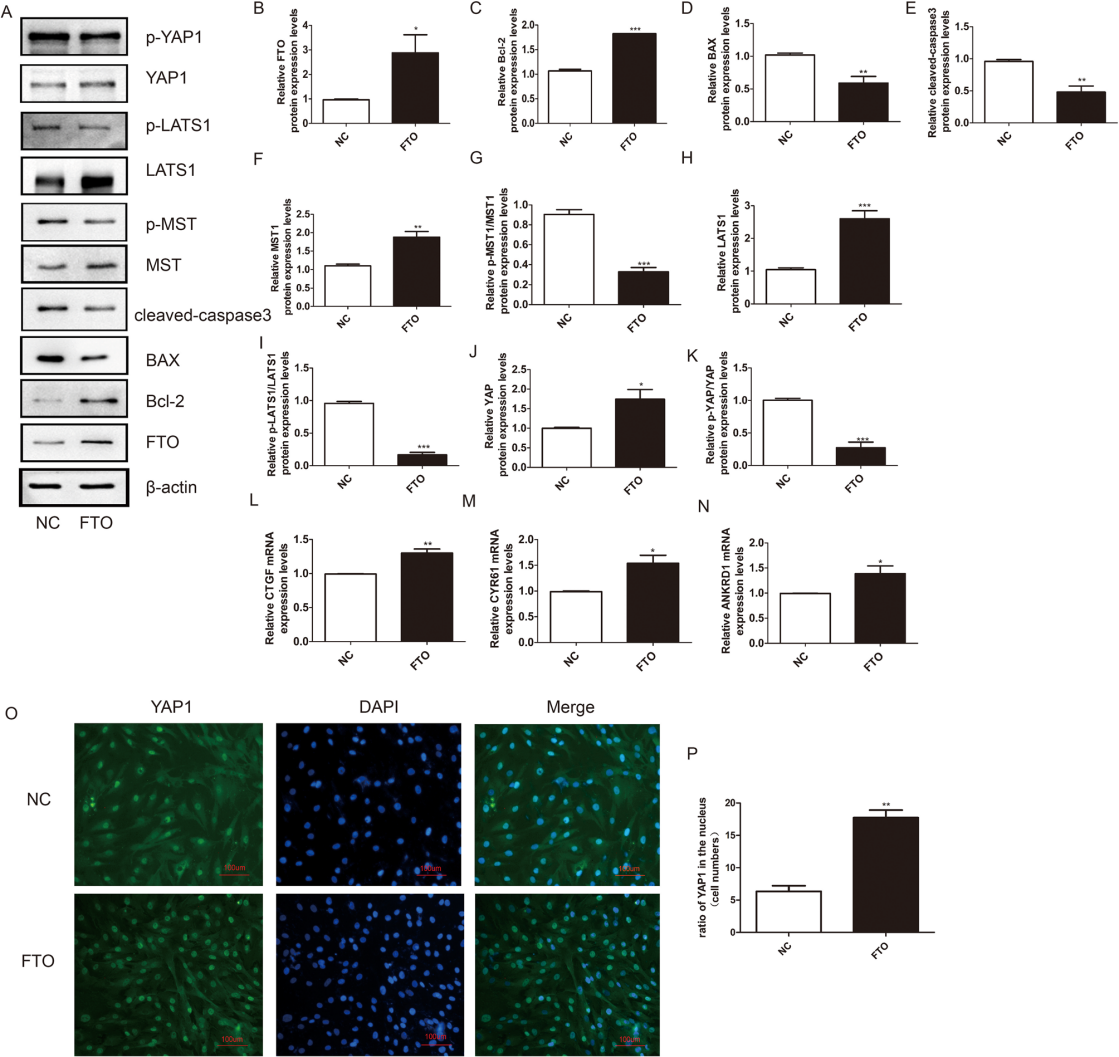
Overexpression of FTO decreased KGN cells apoptosis and disrupted the Hippo/YAP signaling pathway. (**A**) The expression of FTO, Bc-2, BAX, MST, p-MST, LATS1, p-LATS1, YAP1, and p-YAP1 were monitored by western blotting. β-actin was used as a loading control. (**B-K**) Quantitative comparison of expression levels of FTO, Bcl-2, BAX, p-MST1/MST1, p-LATS1/LATS1, p-YAP1/YAP1 in different groups. (**L-N**) The mRNA expression level of CTGF, CYR61, and ANKRD1 were determined by qRT-PCR. GAPDH was used as a loading control. Student’s t-test. The results were showed mean ± SEM from three independent experiments. (α = 0.05, *P < 0.05, **P < 0.01, ***P < 0.001). (O - P) Immunofluorescence assay was performed to determine the effect of FTO on the expression and location of YAP1. (scale bar: 100 μm)

To explore the function of FTO in KGN cell apoptosis and its effect on YAP1 expression, we first designed an FTO plasmid. Overexpression of FTO attenuated the expression of the proapoptotic proteins Bax and cleaved-caspase3 and promoted the expression of the antiapoptotic protein Bcl-2 (Fig. 2A-E). Furthermore, FTO upregulated the protein expression level of YAP1 and decreased the relative ratio of p-YAP1/YAP1. In addition, the expression of Hippo kinase cascade proteins, including MST1 and LATS1, increased markedly, while the ratio of p-MST1/MST1 and p-LATS1/LATS1 desreased (Fig. 2F-K). The downstream effectors of YAP1 were also detected. FTO promoted the expression of the ANKRD1, CYR61, and CTGF mRNAs (Fig. 2L-N). Immunofluorescence staining revealed that the expression of YAP1 in the KGN cell nucleus increased significantly upon FTO overexpression (Fig. 2O - P). Collectively, these results indicated that FTO plays a role as an anti-apoptosis factor through inhibiting the Hippo/YAP signalling pathway and activating the expression of YAP1 in KGN cells.

### Downregulation of FTO promotes KGN cell apoptosis through inhibiting the YAP1 expression

**Fig. 3:**
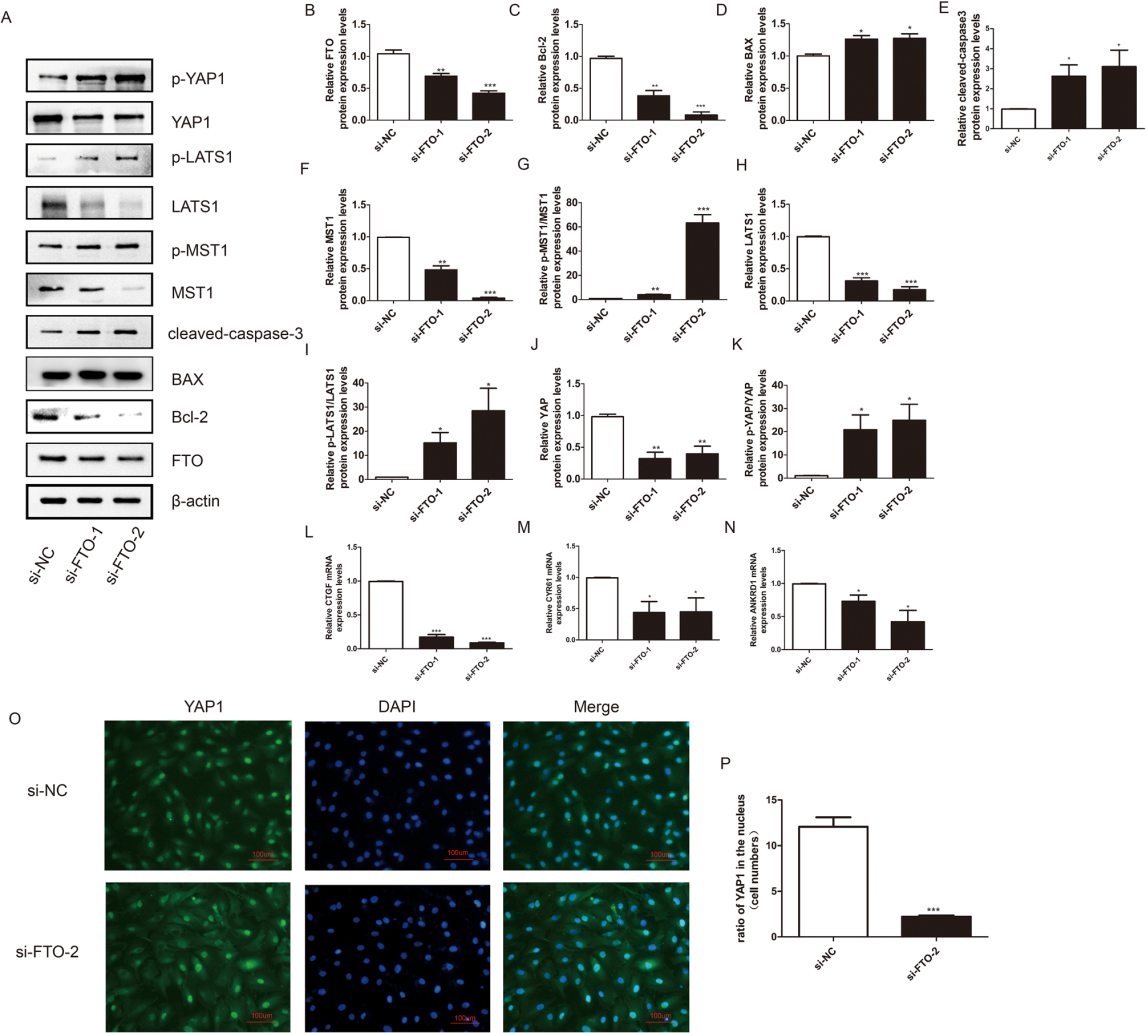
Downregulation of FTO promoted KGN cells apoptosis and activated the Hippo/YAP signaling pathway. (**A**) Western blotting was used to detect the effects of si-FTO on the expression of FTO, Bcl − 2, BAX, MST, p-MST, LATS, p-LATS1, YAP1, and p - YAP1. β-actin was used as a loading control. (**B-K**) Quantitative comparison of expression levels of FTO, Bcl-2, BAX, p-MST1/MST1, p-LATS1/LATS1, p-YAP1/YAP1 in different groups. (**L-N**) The mRNA expression level of CTGF, CYR61, and ANKRD1 were determined by qRT-PCR. GAPDH was used as a loading control. Student’s t-test. The results were showed mean ± SEM from three independent experiments. (α = 0.05, *P < 0.05, **P < 0.01, ***P < 0.001). (**O-P**) Immunofluorescence assay was performed to determine the effect of si-FTO on the expression and location of YAP1. (scale bar: 100 μm)

We then designed siRNAs targeting FTO to further test whether FTO was involved in the cisplatin-induced KGN cell apoptosis by regulating the key regulators of the Hippo/YAP signalling pathway. si-FTO-1 and si-FTO-2 were ultimately selected for further studies due to their high knockdown efficiency. On the basis of our study involving flow cytometry, CCK-8 assay, and EdU assay, we demonstrated that downregulation of FTO promoted granulosa cell apoptosis and inhibited cell proliferation [21]. In this study, downregulation of FTO promoted KGN cell apoptosis by increasing the expression level of BAX and cleaved-caspase 3 and inhibiting the expression of Bcl-2 (Fig. 3A - E). We further detected the key regulators of the Hippo/YAP signalling pathway and found that downregulating FTO expression decreased the protein expression level of YAP1 and promoted the relative ratio of p-YAP1/YAP1. Furthermore, the expression of MST1 and LATS1 was lower in the si-FTO group than in the negative control group. However, the p-MST1/MST1 and p-LATS1/LATS1 ratios were significantly greater (Fig. 3F - K). Immunofluorescence staining revealed that the expression of YAP1 in the KGN cell nucleus decreased markedly in the of si-FTO group (Fig. 3O - P). The downstream effectors of YAP1 were also detected. si - FTO inhibited the mRNA expression level of the ANKRD1, CYR61, and CTGF (Fig. 3L - N). Therefore, we further confirmed that FTO decreased KGN cell apoptosis by regulating the Hippo/YAP signalling pathway.

### Verteporfin reversed the antiapoptotic effects of FTO in cisplatin-induced injured KGN cells

**Fig. 4:**
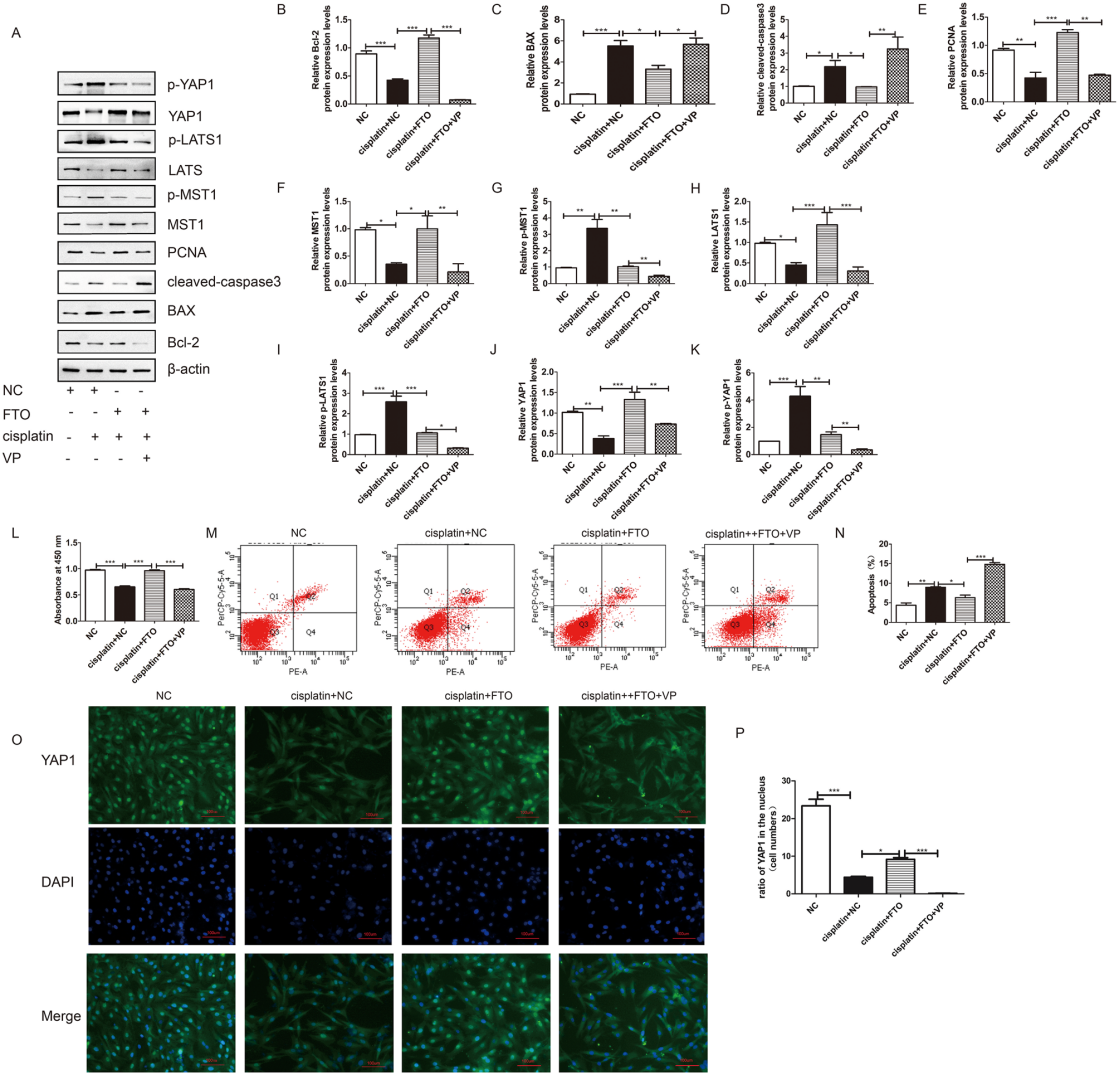
Overexpression of FTO promoted cisplatin-induced injured KGN cells proliferation and decreased their apoptosis via Hippo/YAP signaling pathway, while VP reversed it. (**A**) Western blotting was used to detect the effects of FTO on the expression of Bcl-2, BAX, cleaved-caspase-3, PCNA, MST, p-MST, LATS1, p-LATS1, YAP1, and p-YAP1in injured KGN cells. β-actin was used as a loading control. (**B-K**) Quantitative comparison of expression levels Bcl-2, BAX, cleaved-caspase-3, PCNA, MST, p-MST, LATS1, p-LATS1, YAP1, and p-YAP1 in different groups. (**L**) CCK-8 assay was performed to measure the rate the KGN cells with different treatments. (**M-N**) Flow cytometry assays were performed to determine the apoptosis rate in different groups, and the quantitative comparison was included. One-way ANOVA followed by Tukey’s multiple comparison test. The results were showed mean ± SEM from 3 independent experiments. (α = 0.05, *P < 0.05, **P < 0.01, ***P < 0.001). (**O-P**) Immunofluorescence assay was performed to determine the expression and location of YAP1 in different groups. (scale bar: 100 μm)

Our previous studies demonstrated that FTO could inhibit KGN cell apoptosis by regulating the Hippo/YAP signalling pathway. We wondered whether FTO could reverse cisplatin-induced injured KGN cell apoptosis by regulating the Hippo/YAP signalling pathway. In this study, we found that, compared to the negative control (NC) group treatment, cisplatin treatment promoted KGN cells apoptosis by activating the Hippo/YAP signalling pathway. However, overexpression of FTO in cisplatin-induced injured KGN cells promoted proliferation and decreased apoptosis (Fig. 4). This change was accompanied by the downregulation of BAX and cleaved-caspase 3 and upregulation of PCNA and Bcl-2 (Fig. 4A - E). Furthermore, compared to the cisplatin-treated group, the expression of MST1, LATS1, and YAP1 in the FTO treated group was significantly greater, while the expression of p-MST1, p-LATS1, and p-YAP1 was significantly lower (Fig. 4F - K).

As the core effector and final step of the Hippo/YAP signalling pathway, YAP/TAZ can regulate cell proliferation and chemoresistance [34]. Verteporfin (VP) can target and block the YAP-TEAD complex, and the potential side effects of VP on the upstream were smaller [34]. In the present study, we found that VP reversed the pro-proliferative and anti-apoptotic effects of FTO on injured KGN cells (Fig. 4L-N). Furthermore, western blot analysis indicated that, compared to that in the FTO treated group, the expression of YAP1 in the VP-treated group was visibly lower. Immunofluorescence also showed that FTO significantly increased the expression of YAP1 in the injured KGN cell nucleus, while in the VP-treated group, YAP1 was hardly detected in the nucleus (Fig. 4O - P). Furthermore, after the VP treatment, the expression levels of MST and LATS were also decreased. This was consistent with the findings of previous studies [35]. VP can inhibit not only YAP/TAZ but also autophagy [36]. Whether other signaling molecules are involved, and whether the side effects of these other signalling pathways on the upstream molecules are larger in our injured KGN cells need further exploration.

CCK-8 and flow cytometry analyses showed that FTO promoted injured KGN cell proliferation and decreased injured KGN cell apoptosis significantly, while VP treatment reversed these changes (Fig. 4L-N).

### Verteporfin increased the proapoptotic effects of si-FTO on injured KGN cells

**Fig. 5:**
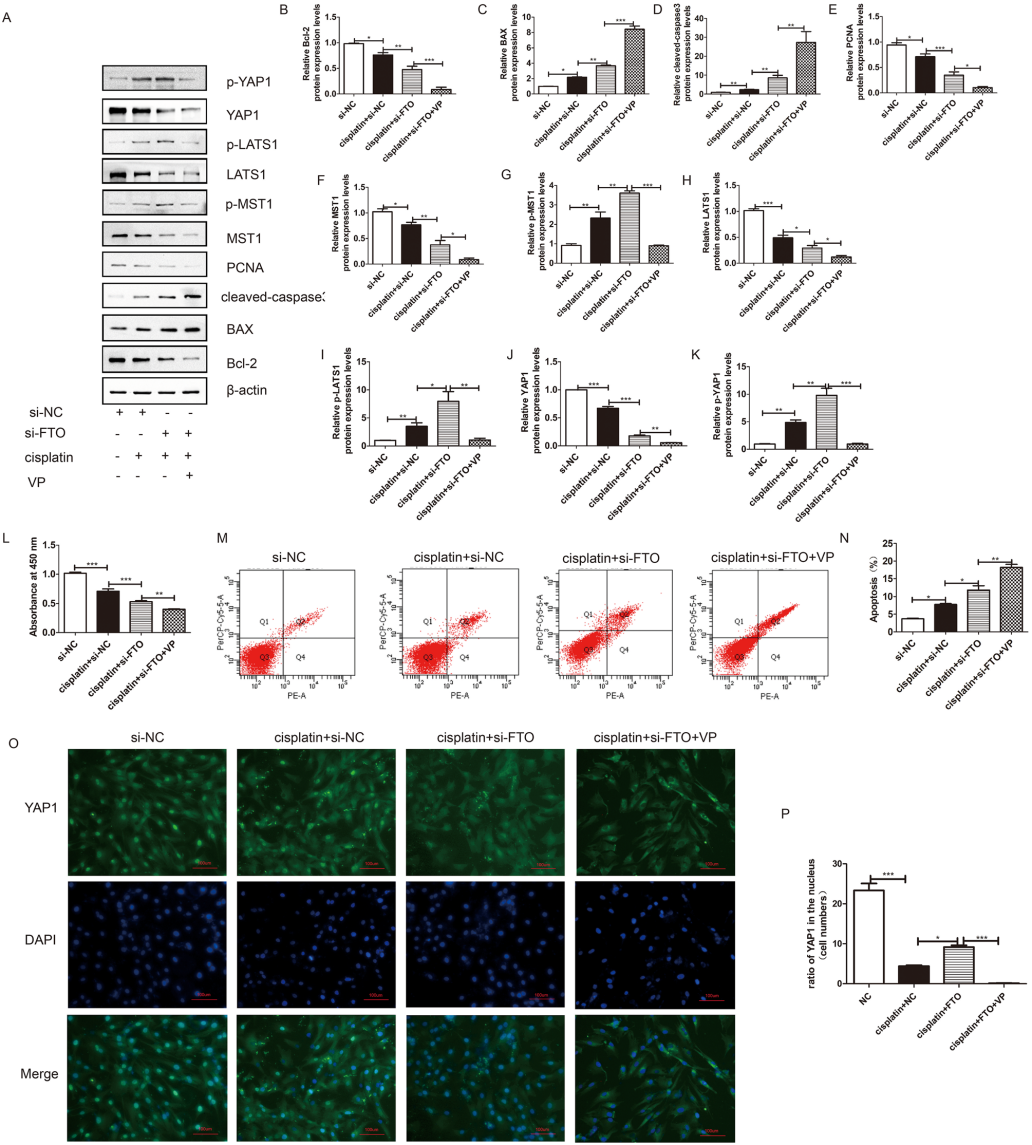
Downregulation of FTO promoted the cisplatin-induced KGN cells injury, and VP could aggregate it. (**A**) Western blotting was used to detect the effects of si -FTO on the expression of Bcl-2, BAX, cleaved-caspase-3, PCNA, MST, p-MST, LATS1, p-LATS1, YAP1, and p-YAP1in injured KGN cells. β-actin was used as a loading control. (**B-K**) Quantitative comparison of expression levels Bcl-2, BAX, cleaved-caspase-3, PCNA, MST, p-MST, LATS1, p-LATS1, YAP1, and p-YAP1 in different groups. (**L**) CCK-8 assay was performed to measure the rate the KGN cells with different treatments. (**M-N**) Flow cytometry assays were performed to determine the apoptosis rate in different groups, and the quantitative comparison was included. One-way ANOVA followed by Tukey’s multiple comparison test. The results were showed mean ± SEM from 3 independent experiments. (α = 0.05, *P < 0.05, **P < 0.01, ***P < 0.001). (**O-P**) Immunofluorescence assay was performed to determine the expression and location of YAP1 in different groups. (scale bar: 100 μm)

We further explored the effects of downregulating of FTO expression on cisplatin-induced injured KGN cells. The results showed that downregulation of FTO in cisplatin-induced injured KGN cells promoted cisplatin-induced apoptosis through activation of the Hippo/YAP signalling pathway. Western blotting indicated that cisplatin treatment increased KGN cell apoptosis by increasing the expression of BAX and cleaved-caspase 3 and inhibiting the expression of PCNA and Bcl-2 (Fig. 5A - E). Accompanied by the downregulation of MST1, LATS1, and YAP1, and the upregulation of p-MST1, p-LATS1, and p-YAP1 (Fig. 5F-K). Downregulation of FTO in injured KGN cells promoted the proapoptotic effects of cisplatin by inhibiting the key regulator of the Hippo/YAP signalling pathway (Fig. 5A-K). We also found that VP could increase the effects of si-FTO on injured KGN cells by inhibiting the expression of YAP1 (Fig. 5A-K). Immunofluorescence also showed that si-FTO significantly decreased the expression of YAP1 in the injured KGN nucleus. In the VP group, YAP1 was hardly detected in the nucleus (Fig. 5O - P).

In addition, CCK-8 and flow cytometry analyses showed that, compared with that in the cisplatin treatment group, the downregulation of FTO promoted the apoptosis of injured KGN cells and inhibited their proliferation. Moreover, VP treatment aggravated the effects of si-FTO (Fig. 5L - N).

## Discussion

In this study, we found that the FTO/YAP1 axis plays a vital role in protecting granulosa cells from cisplatin-induced cytotoxicity. Overexpression of FTO disrupted the Hippo signalling pathway, decreased YAP1 phosphorylation, and subsequently increased YAP1 translocation to the nucleus, promoting the proliferation of KGN cells, which play an essential role in the development of follicles.

FTO is a key m^6^A demethylase that can regulate cell proliferation, differentiation, and the sensitivity to radiotherapy and chemotherapy [32]. Furthermore, FTO may function as a novel biomarker for POF [20]. On the basis of our previous study, FTO expression was decreased significantly in cisplatin-induced injured KGN cells and in the follicles of cisplatin-induced POF mice [37]. Additional, overexpression of FTO reserved the proliferation ability of cisplatin-induced in KGN cells. In this study, we found that in the cisplatin-induced injured KGN cells, the expression of FTO and YAP1, a key regulator of the Hippo signalling pathway, decreased in a concentration-dependent manner. However, the mechanism underlying their correlation remains unclear.

Various studies have demonstrated that the Hippo signalling pathway plays important roles in the ovary. Abnormalities in the Hippo/YAP signalling pathway are closely related to a variety of ovarian diseases, such as premature ovarian insufficiency (POI), ploycystic ovary syndrome (PCOS), and epithelial cell tumours of the ovary [38]. As the core regulator of the Hippo signalling pathway, LATS1 is expressed in normal ovarian epithelia, the endometrium, and fallopian tubes [39]. Moreover, the inactivation of LATS1 in granulosa cells causes a loss of cell morphology, function, and gene expression [30]. Furthermore, LATS1 knockout mice were subfertile [40]. YAP1, which functions as the major effector of the Hippo signaling pathway, is expressed in the ovarian granulosa cells and plays a vital role in regulating the development of follicles. Knockdown of YAP1 in granulosa cells promoted cell apoptosis, disrupted the ovarian follicle development in mice, and resulted in subfertility [26]. Previous studies have suggested that there are four m^6^A motifs on YAP1 mRNA 3’-UTR, and FTO could directly bind to these domains to increase the stability of YAP1 mRNA [32]. Consistence with these findings, in the present study, we found that overexpression of FTO disrupted the Hippo signalling pathway, promoted the nuclear translocation of YAP1, and increased the expression of downstream targets of YAP1, such as CTGF, CYR61, and ANKRD1, leading to the proliferation of KGN cells. Additionally, FTO could reverse the damages of cisplatin on KGN cells in a YAP1-dependent manner. These results demonstrated that FTO disrupted the Hippo signalling pathway, especially through interaction with YAP1, accelerated YAP1 transport to the nucleus, and promoted the functional repair of the injured KGN cells induced by cisplatin.

This study has a number of limitations. For example, the data would be more convincing if ovarian tissues from cisplatin-induced POI and POF patients could be collected to determine the differences in the expression levels of FTO and molecules in the Hippo/YAP signalling pathway. Due to ethical and moral limitations, we had no access to obtain the samples. Furthermore, overexpression of the FTO gene cannot be achieved in humans. Future studies should focus on the exploring FTO activator and inhibitor and observing the effects on the ovaries.

Cisplatin is not only a chemotherapeutic agent but also an anti-metabolic agent [41]. FTO is a gene that plays an important role in regulating metabolism and is involved in the glycolipid metabolism by regulating different genes [42]. And YAP could promote glycolysis, lipogenesis, and glutaminolysis to maintain cellular homeostasis [43]. As the main energy source for follicle development, glycolysis occurs and is enhanced in granulosa cells during the follicle activation [44]. We speculate that the FTO/YAP axis may protect granulosa cells from cisplatin-induced injury by regulating the metabolism of injured granulosa cells. In our future study, we will pay more attention to this part.

In conclusion, our study found that in cisplatin-induced injured KGN cells, the expression of FTO and YAP1 decreased in a dose-dependent manner. Overexpression of FTO disrupted the Hippo signalling pathway and promoted YAP1 translocation to the nucleus. Downregulation of FTO had the opposite effect. Furthermore, for the first time, our studies demonstrated that FTO/YAP1 axis plays important role in regulating the cisplatin-induced injured KGN cell proliferation and apoptosis.

### Electronic supplementary material

Below is the link to the electronic supplementary material.

**Supplementary Material 1**: IgG was used as the negative control to test the specificity of the YAP1 antibody

**Supplementary Material 2**: RT-PCR and Western blotting assay were applied to test the transfection efficiency of si-FTO in KGN cells

## Data Availability

The data that support the findings of this study are available in the methods and material of this article.
