# Supplementary figures and images for: FTO attenuates the cytotoxicity of cisplatin in KGN granulosa cell-like tumour cells by regulating the Hippo/YAP1 signalling pathway
Source: J Ovarian Res. 2024 Mar 15;17:62. doi: 10.1186/s13048-024-01385-5 (PMC10941382; doi:10.1186/s13048-024-01385-5)

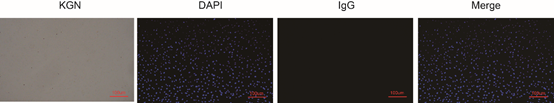

Supplement: Supplementary file 1 — Supplementary Material 1: IgG was used as the negative control to test the specificity of the YAP1 antibody [file 13048_2024_1385_MOESM1_ESM.jpg]

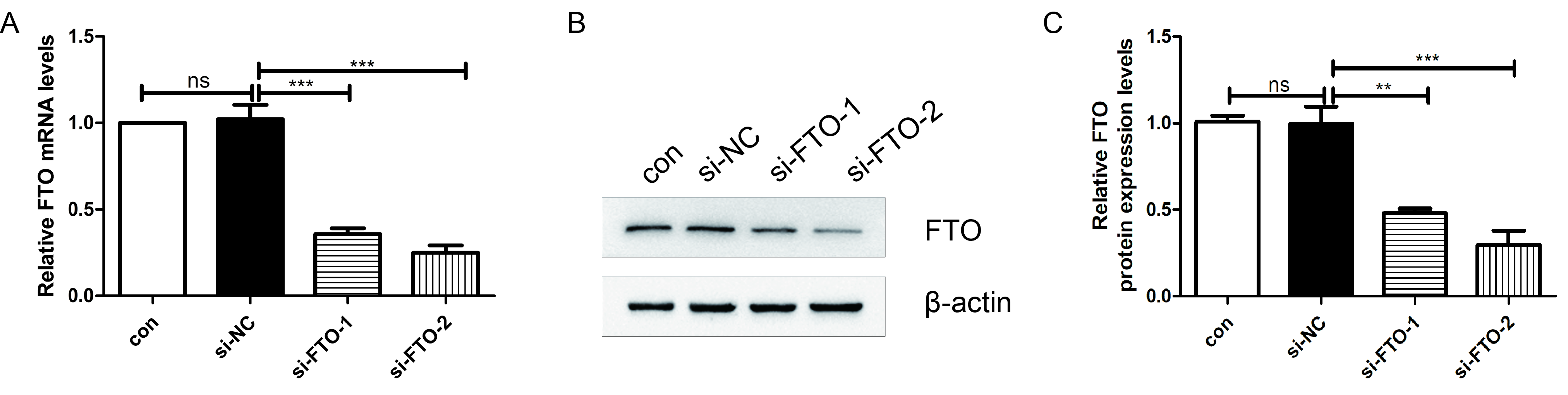

Supplement: Supplementary file 2 — Supplementary Material 2: RT-PCR and Western blotting assay were applied to test the transfection efficiency of si-FTO in KGN cells [file 13048_2024_1385_MOESM2_ESM.tif]
